# Meeting of the Ohio State Dental Society

**Published:** 1884-12

**Authors:** 


### EDITORIAL.

### Meeting of the Ohio State Dental Society.

The regular annual meeting of this body was held in Columbus October 29th, and continued two days. There was a reasonably good attendance, though many of the older members were absent. After the usual routine business was disposed of, the cases of Drs. Warner and Garris, who were under suspension since last year for unprofessional conduct, were taken up. In order to bring the matter before the Society, a resolution was offered to remove the suspension. Evidence was presented, however, that the parties had repeated the offence since their suspension, and they evinced no disposition to make amends, or place themselves in a proper position in regard to the matter. They seemed to have no just appreciation of the character of the offence with which they were charged, and which was clearly proven. Quite a strong defence was made in their behalf, and considerable argument was had, pro and con, but, after due consideration, a resolution of expulsion was adopted, with but two dissenting votes. This decision was manifestly made with much regret on the part of the majority of the members, for the gentlemen in question had many friends in the Society. They are pleasant, genial gentlemen, and ought to have been wise enough to have avoided the rock upon which they ran. It is no light matter to receive such a sentence of disapproval of a course of conduct by so large a number of compeers. The Society, however, realized the fact that it could take no other course without stultifying itself. After this business was completed, two or three papers were read which elicited some discussion. A large share of the time of the meeting, however, was occupied in the discussion of subjects not calculated for improvement or harmony in the Society. The Society has labored under much embarrassment in this respect for the last three or four years, and seems to be growing worse each year; so, near the time of adjournment, the following resolution was offered:

*Resolved*, That upon the final adjournment of this session, the Association shall adjourn *sine die*, and that at that time the organization shall be dissolved.

This resolution was unanimously adopted. It was decided not to publish the transactions in a separate volume, as heretofore, but it was requested that they be published in the DENTAL REGISTER, and the *Ohio State Journal of Dental Science*. After some further formal proceedings the Association adjourned, according to the above resolution. A determination was soon afterward evinced by many of the old members that the profession should not be long without a State Society, and, so, within a few hours afterward some twenty, or more, members of the profession were called together to consider what should be done in the future. The opinion was unanimously expressed that a reorganization should be effected, and, after full consideration, this was done; in the accomplishment of which officers were elected, and articles of incorporation were drawn up, signed and placed upon the records of the State. A committee was appointed to draft a constitution and by-laws, to be presented at the next meeting, which will be held at Chillicothe, Ohio, on the last Wednesday of October, 1885, when it is hoped that a meeting will be held, free from the difficulties that attended the old Society, and that under the new organization good and efficient work may be accomplished.

The proceedings of the reorganization are given on page 606 of this No. of the REGISTER.

